# Vitality Forms Processing in the Insula during Action Observation: A Multivoxel Pattern Analysis

**DOI:** 10.3389/fnhum.2016.00267

**Published:** 2016-06-09

**Authors:** Giuseppe Di Cesare, Giancarlo Valente, Cinzia Di Dio, Emanuele Ruffaldi, Massimo Bergamasco, Rainer Goebel, Giacomo Rizzolatti

**Affiliations:** ^1^Department of Neuroscience, University of ParmaParma, Italy; ^2^Department of Cognitive Neuroscience, Faculty of Psychology and Neuroscience, Maastricht UniversityMaastricht, Netherlands; ^3^Department of Psychology, Università Cattolica del Sacro CuoreMilan, Italy; ^4^The Laboratory of Perceptual Robotics (PERCRO), Institute of Communication, Information and Perception Technologies (TeCIP), Scuola Superiore Sant’AnnaPisa, Italy; ^5^Brain Center for Social and Motor Cognition, Italian Institute of Technology (IIT)Parma, Italy; ^6^Istituto di Neuroscienze, Consiglio nazionale delle RicercheParma, Italy

**Keywords:** vitality forms, insula cortex, MVPA, social interaction, action understanding

## Abstract

Observing the style of an action done by others allows the observer to understand the cognitive state of the agent. This information has been defined by Stern “vitality forms”. Previous experiments showed that the dorso-central insula is selectively active both during vitality form observation and execution. In the present study, we presented participants with videos showing hand actions performed with different velocities and asked them to judge either their vitality form (gentle, neutral, rude) or their velocity (slow, medium, fast). The aim of the present study was to assess, using multi-voxel pattern analysis, whether vitality forms and velocities of observed goal-directed actions are differentially processed in the insula, and more specifically whether action velocity is encoded *per se* or it is an element that triggers neural populations of the insula encoding the vitality form. The results showed that, consistently across subjects, in the dorso-central sector of the insula there were voxels selectively tuned to vitality forms, while voxel tuned to velocity were rare. These results indicate that the dorso-central insula, which previous data showed to be involved in the vitality form processing, contains voxels specific for the action style processing.

## Introduction

The observation of goal-directed actions done by another individual allows the observer to achieve, typically, an immediate comprehension of *what* that individual is doing (see Rizzolatti et al., 2014). Besides goal, the observation of a goal-directed action allows the observer to understand, on the basis of *how* the action is performed, the psychological state of the agent. It also provides, in the case of interpersonal actions, an appraisal of the affective/communicative qualities underlying the relation between the agent and the action recipient. These aspects of action comprehension have been named by Stern (1985, 2010) “vitality affects” or “vitality forms”.

According to Stern (1985, 2010), the appraisal of vitality forms depends on the kinematics properties of the observed movement (time, space, force, direction). These movement properties create a particular experience that reflects the affective/communicative state of the agent. The capacity to express and understand the vitality forms is already present in infants. These abilities denote a primordial way to relate and to understand others and represent a fundamental constitutive element of interpersonal relations (Stern, 1985, 2010; Trevarthen, 1998; Trevarthen and Aitken, 2001).

In a previous functional magnetic resonance imaging (fMRI) study (Di Cesare et al., 2013) an attempt was done to define the brain areas specifically involved in vitality form processing by comparing brain activations during vitality forms judgment with respect to the activations observed during goal understanding of the same action. The results showed that a key structure involved in vitality forms processing was the dorso-central sector of the insular cortex. These data were confirmed by a further experiment in which participants had to judge the vitality form of an action, imagine to perform it, and to execute it (Di Cesare et al., 2015).

The aim of the present study was to assess using multi-voxel pattern analysis (MVPA, Edelman et al., 1998; Haxby et al., 2001; Cox and Savoy, 2003; Haynes and Rees, 2005; Norman et al., 2006; Kriegeskorte et al., 2006; Kriegeskorte and Bandettini, 2007) whether observing an action performed with different velocities will produce in the insula distinct activation patterns according as to whether the participants had to judge the action velocity or pay attention to its vitality form. Videos showing actions performed with three velocities were selected and presented to the participants. These velocities corresponded to fast/rude (1.06 m/s), medium/neutral (0.57 m/s) and slow/gentle (0.38 m/s) velocities and vitality forms, respectively. These velocities were selected on the basis of a preliminary behavioral experiment in which participants observed actions performed with 12 different velocities and had to judge them as very rude/very fast, rude/fast, neutral/medium, gentle/slow, and very gentle/very slow, according to the instructions.

The MVPA analysis showed the presence of a large number of discriminative voxels with positive sign, that is exhibiting a statistically significant preference for vitality, relative to velocity while discriminative voxels exhibiting a statistically significant preference for velocity were few. The insula sector containing voxels with positive sign corresponded to the dorso-central sector of the insula.

These findings indicate that the dorso-central insula does not encode velocity parameters, but use this information to trigger the region located in the dorso-central insula that previous data showed to be involved in the control of the action style (Di Cesare et al., 2015). These data provide strong support for the view that insula transforms the physical aspects of an observed action in a communicative/affective construct (vitality form). In virtue of this mechanism the observer is able to understand the internal state of others.

## Materials and Methods

### Behavioral Study

#### Subjects

Eighteen healthy right-handed participants (mean age = 23.5 years, SD = 1.85 years) took part to the behavioral study. All participants had normal or corrected-to-normal visual acuity. They gave their written informed consent to the experimental procedure, which was approved by the Local Ethics Committee (Parma, Italy).

#### Stimuli and Experimental Design

**Figure 1:**
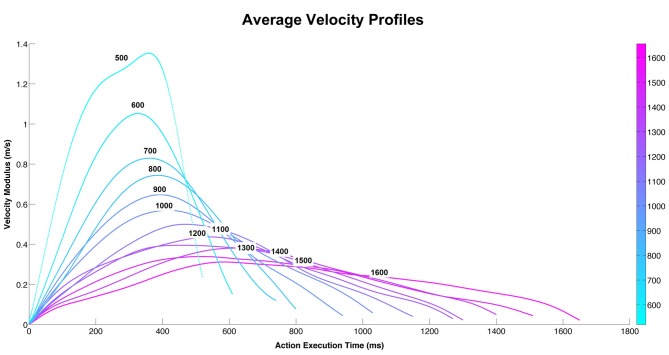
**The graph depicts the average velocity profiles of the action performed by the male actor during 12 different execution times.** Each velocity curve represents the main velocity used by the actor to perform the action (pass an object towards the other actor) using three different objects (bottle, can, jar) at 12 different execution times.

**Figure 2:**
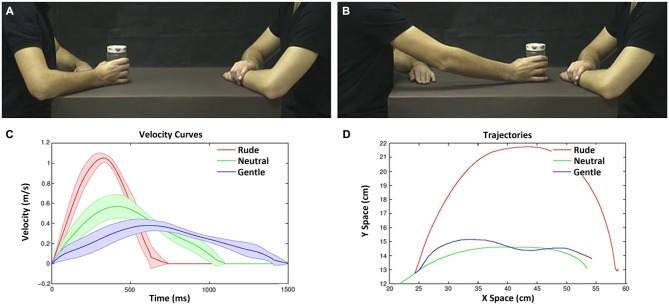
**Example of video-clips as viewed by the participants in the experiment (A,B) and their physical properties (C,D).** Frame representing an action with the object in the start position **(A)**; frame representing the same action in the end position **(B)**. Velocity and trajectory profiles of the actions performed by the male actor (move a bottle, can and jam) with three vitality forms. Graph depicts the velocity profiles (*Y* axis) and duration (*X* axis) **(C)**. Graph depicts the action trajectories (gentle, blue line; neutral, green line; rude, red line; **D**).The variance among objects is represented by the lines boundary.

The participants were shown video-clips representing two actors, one of which moved an object (a bottle, a can, or a jar) with his right hand towards the other actor. All three actions were performed with 12 different velocities (Figure 1). In all videos, the actor started from the same initial position and reached the same final position. Figures 2A,B show the action performed with a jar. Each video lasted 2 s. A total of 36 stimuli were presented (3 objects × 12 velocities). The experimental design was a 2 × 12 factorial with two levels of task (*vitality, velocity*) and twelve levels of velocities (execution time from 500 ms to 1600 ms).

#### Paradigm and Task

The experiment consisted of four experimental sessions. To avoid possible influences of the velocity task on the vitality task, we presented the vitality task before the velocity one. Thus, in the first two sessions, participants were instructed to judge the vitality forms of the actions, judge them as “very rude”, “rude”, “neutral”, “gentle”, or “very gentle” using a five point scale (*vitality task*). In the third and fourth sessions, participants were asked to evaluate the velocity of the same stimuli and to judge them as “very fast”, “fast”, “medium”, “slow”, and “very slow” using again a five point scale (*velocity task*). Before the first and the third experimental session, participants underwent a training session (vitality training, before to start the session 1; velocity training, before to start the session 3), with different stimuli from those used during the experiment, to familiarize with the experimental procedures and tasks.

Using E-Prime Software, a total of 36 stimuli were presented for the vitality and velocity tasks (3 actions, i.e., move a bottle, move a jar, move a can, each one presented with 12 different velocity). Each action was presented 10 times per task. Each experimental session consisted of 180 trials presented in a randomized order. Each session lasted about 10 min, the whole experiment lasting about 45 min.

The velocity profile of each action was assessed by placing a reflective marker on the object using 3D motion capture system (Vicon OMG, UK). In particular, six infrared cameras (MX2 model) recorded the position occupied by the marker in the 3D space for each action performed by the actor with the object. After recording with Vicon Nexus at 100 Hz, all recorded data were used to perform a kinematic analysis, using MATLAB (The Mathworks, Natick, MA, USA) Software.

The 36 stimuli (3 objects × 12 velocities) used in the experiment have been compared by means of the Dynamic Time Warp (DTW; Berndt and Clifford, 1994; Ding et al., 2008) metrics that allows to take into account the little differences in duration of the trajectories. The DTW allows to measure the distance between two time series that have different duration by finding the correspondences between points in the time-series by means of a dynamic programming approach. This metrics has been applied to the modulus of the velocity of each trajectory (and on vx, vy, vz independently) and it produces a 36×36 matrix of pairwise distances. The distance matrix had been analyzed for understanding if, for every duration level, the distance among the objects inside each level of velocity, is less than the ones of other duration levels. The results of this analysis showed that there was no difference between the three objects. For this reason we grouped the three objects and calculated the velocity average profiles of the three objects (bottle, can, jar; Figure 1).

### fMRI Studies

#### Participants

Sixteen healthy right-handed volunteers [8 females (mean age = 24.1 years, SD = 2 years, range = 21–28 years) and 8 males (mean age = 24.4 years, SD = 2.18 years, range = 22–29 years)] participated in the experiment. All participants had normal or corrected-to-normal visual acuity. They gave their written informed consent to the experimental procedure, which was approved by the Local Ethics Committee (Parma, Italy).

#### Experimental Design and Stimuli

The experimental design was a 2 × 3 factorial with two levels of task (*vitality, velocity*) and three levels of vitalities/velocities (*gentle/slow, neutral/medium, rude/fast*). During the experiment, participants were shown video-clips representing two male actors, one of which (the one sitting on the left side of the screen) performed an action towards the other actor using his right hand (Figures 2A,B). To keep the observer’s attention, the action was executed using three different objects (move a bottle, a can, a jar). All actions were performed using three different velocities (execution times: 600, 1000, 1400 ms; mean velocity: 1.06, 0.57, 0.38 m/s; see Figure 2C). These stimuli were selected on the basis of a previous behavioral experiment. They mostly corresponded to fast/rude, medium/neutral and slow/gentle velocity/vitality judgments (see also Supplementary Figure 1). In all videos, the actor started from the same initial position (Figures 2A,D) and reached the same final position (Figures 2B,D). Each video lasted 2 s. A total of nine stimuli were shown (3 *objects* × 3 *execution times*).

#### Paradigm and Task

Participants lay in the scanner in a dimly lit environment. The stimuli were viewed via digital visors (VisuaSTIM) with a 500,000 px × 0.25 square inch resolution and horizontal eye field of 30°. The digital transmission of the signal to the scanner was via optic fiber. The software E-Prime 2 Professional (Psychology Software Tools, Inc., Pittsburgh, PA, USA, http://www.pstnet.com) was used both for stimuli presentation and the recording of participants’ answers.

The experiment was composed of four functional runs (2 for *vitality* task, 2 for *velocity* task). Similarly to the behavioral task, to avoid possible biases elicited by the velocity condition on the vitality form judgment, we presented the vitality form condition before the velocity condition. Thus, in the first two runs, we presented participants with video clips and asked them to pay attention to the style of the action (*vitality* task). In the last two runs, we presented participants with the same video clips and asked them to pay attention to action velocity (*velocity* task). A fixation cross was introduced in each video to restrain eye movements.

Every run started with a white fixation cross, positioned at the center of a black screen for 12 s. Each experimental trial presented a single video-clip for 2 s followed by a jittered interval (fixation cross) ranging 12–16 s. In 10% of cases, after 500 ms from video viewing, the participants were cued presenting a task related question lasting 2.5 s. During this time, they had to provide an explicit response to the stimuli (catch trials). More specifically, during the view of the question cue (2.5 s), the participants had to indicate, on a response box placed inside the scanner, whether the observed video was rude/fast, neutral/medium, gentle/slow according to the task-type. In total, participants viewed 50 video-clips (45 experimental trials, 5 catch trials) for each run, presented in a randomized order. Each functional run lasted about 14 min.

Before the first and the third experimental session, participants underwent a training session (vitality training, before to start the session 1; velocity training, before to start the session 3), with different stimuli from those used during the experiment, to familiarize with the experimental procedures and tasks.

#### fMRI Data Acquisition

Anatomical T1-weighted and functional T2*-weighted MR images were acquired with a 3 Tesla General Electrics scanner equipped with an 8-channel receiver head-coil of the Department of Neuroscience of University of Parma. Functional images were acquired using a T2*-weighted gradient-echo, echo-planar imaging (EPI) pulse sequence (acceleration factor asset 2, 37 interleaved transverse slices covering the whole brain, with a repetition time (TR) time of 2000 ms, echo time (TE) = 30 ms, flip-angle = 90°, field of view (FOV) = 205 × 205 mm^2.^ inter-slice gap = 0.5 mm, slice thickness = 3 mm, in-plane resolution 2.5 × 2.5 × 2.5 mm^3^). Each scanning sequence comprised 416 interleaved volumes. Before the third functional run, to allow participants to rest, a high-resolution inversion recovery prepared T1-weighted anatomical scan was acquired for each participant (acceleration factor arc 2, 156 sagittal slices, matrix 256 × 256, isotropic resolution 1 × 1 × 1 mm^3^, TI = 450 ms, TR = 8100 ms, TE = 3.2 ms, flip angle 12°).

### Statistical Analysis

#### Univariate Analysis

Data analysis was performed with Brain Voyager QX (Brain Innovation). The raw images were pre-processed in Brain Voyager QX performing the following steps: sinc-interpolated slice-time correction, 3D motion correction to correct small head movements, temporal high-pass filtering to remove low frequency components up to seven cycles for time course. Functional slices were then coregistered to the anatomical volume and subsequently transformed into Talairach space. All individual brains were segmented at gray/white matter boundary using a semiautomatic procedure based on intensity values implemented in Brain Voyager QX. We applied a minimal amount of spatial smoothing to reduce the residual effects of head movement (1-mm full-width half-maximum (FWHM) isotropic Gaussian kernel).

Data were analyzed using a random-effects model (Friston et al., 1999), implemented in a two-level procedure. In the first level, single-subject fMRI responses were modeled in a general linear model (GLM) by a design-matrix comprising the onsets and durations of each event according to the experimental task for each functional run.

In the experiment, at the first level, for the task *vitality* we modeled four regressors as follows: *Rude, Neutral, Gentle, and Response*; for the task *velocity* we modeled other four regressors as follows: *Fast, Medium, Slow, and Response*. The single video of each trial was modeled as a mini epoch lasting 2 s. The *Response* for the first level analysis was modeled with 2.5 s starting from the question was presented. In the second level analysis (group-analysis), corresponding contrast images of the first level for each participant were entered in a random effects GLM (Friston et al., 2002). This model was composed of six regressors (*Fast, Medium, Slow, Rude, Neutral, Gentle*) and considered the pattern of activation obtained for each level in the two tasks (*vitality*, *velocity*) vs. implicit baseline.

Within this model, we assessed activations associated with each task vs. implicit baseline (*p*_FDR_ < 0.05). This model did not reveal significant main effect of task (vitality *vs*. velocity), levels (*Rude vs. Gentle*, *Neutral vs. Gentle*, *Rude vs. Neutral*), or interaction.

The location of the activation foci was determined in the Talairach coordinates system. Those cerebral regions for which maps are provided were also localized using the Talairach Client Software (version 2.4.3).

#### Testing for Task-Complexity: Behavioral Analysis

Our contrast of interest, vitality vs. velocity could have reflected some effects associated with task presentation order such as a possible fatigue effect. To test this possibility, we carried out a further analysis, based on the responses given by the participants during the scanning sessions when presented with the catch trials, i.e., those trials in which the participants were required to give an explicit response on the presented videos, indicating if they were rude, neutral, gentle in terms of vitality form (vitality task) or fast, medium, slow (velocity task). Ten responses were recorded for each task for each participant. The dependent variable was the percent of correct responses (“hits”). On these behavioral data, a GLM analysis was carried out.

#### Multivoxel Pattern Analysis in the Insula

A multivoxel pattern analysis was then carried out to assess possible different activation patterns in the insula in response to vitality form (rude, neutral, gentle) and velocity (fast, medium, slow). We decoded multivariate pattern of BOLD activation using support vector machine (SVM) classifiers based on stimulus perception. On the basis of our previous results (Di Cesare et al., 2013, 2015), we tested the activation pattern characterizing the insular cortex in response to different action vitality forms (Rude, Neutral, Gentle) compared to their velocities (Fast, Medium, Slow). We built two regions of interest (ROIs), one at level of the left insula (size of 1533 voxels) and one at the level of the right insula (size of 1346 voxels). In order to build the two ROIs, we drew a line between the border of the insula and the parietal, frontal and temporal opercula cortices, which were all excluded from the ROIs. To make sure that each drawn point belonged to the insula, for each slice we checked the coordinates of 8 different border points with Talairach coordinates (Talairach Client—V.2.4.3). We also built two control ROIs, one (CTRL 1) at level of the white matter (size of 500 voxels, coordinates −28, −41, 26) and the other (CTRL 2) at level of Broadman Area 21 (BA 21) (size of 750 voxels, coordinates −48, −4, −22). The control ROIs served to test results reliability as a function of the multivoxel pattern model. All ROIs were built on the mean anatomical structure of the participants. We estimated the response of every voxel in each trial by fitting a standard hemodynamic model to each voxel. The patterns of activation related to each given trial consisted of the set of beta (% change) values associated with one of the six predictors (*task × levels model*) for all voxels considered in the analysis. The Inter-Stimulus-Interval ranged from 6 to 8 TRs (12–16 s). For each trial, one pre-onset volume and 5 post-onset volumes were used to model the signal.

Since the multivoxel pattern model required a comparison between tasks that were presented in separate runs (*vitality task*: runs 1, 2; *velocity task*: runs 3.4), we performed a cross-validation scheme considering alternate runs (1.3; 2.4; 2.3; 1.4), dividing them in two different groups (training runs and testing runs). More specifically, we trained linear SVMs on the training datasets (e.g., from runs 1.3) and evaluated the generalization of the model to new data (the test datasets example e.g., from runs 2.4). This procedure was repeated for four possible combinations (1.3 vs. 2.4; 2.4 vs. 1.3; 2.3 vs. 1.4; 1.4 vs. 2.3). To ascertain that this difference cannot be explained by global effects such as amplitude differences between runs, we conducted an additional ROI analysis considering only the voxels in the left and right insula, testing for univariate differences between vitality and velocity runs.

We reported accuracies for the classification of new trials. Using balanced datasets for training and testing (15 trials for each level, *rude/neutral/gentle*; 15 trials for each level, *fast/medium/slow*), we expected a rate higher than 50% (expected chance level, obtained with 1000 permutations, see Figure 4) for each different contrast (*rude vs. fast, neutral vs. medium, gentle vs. slow*). The significance of this difference was assessed by means of non-parametric Wilcoxon sign-rank one-sided test (α = 0.05).

To visualize the spatial activation patterns that were used for classification and to assess consistency across participants, group discriminative maps were created. For each participant, these maps indicated the locations that contributed the most to the discrimination of conditions. After using the linear SVM we ranked the features (i.e., voxels) according to their contribution to the discrimination at each individual map level and selected the peaks through thresholding. For each participant, we selected the 50% most discriminative voxels and created group discriminative maps representing the overlap between the 16 participants. To calculate a *p*-value for each voxel, we used a Monte Carlo simulation, where we randomly selected 50% (or 35%) voxels from each subject, and determined the overlap between subjects, under the assumption that the spatial maps are completely unrelated. To account for the multiple tests performed in creating these maps, we thesholded the maps using false discovery rate (Benjamin and Hochberg, 1995, with *q* = 0.05), resulting in at least 10 of 16 participants. It is worth noting that we obtained the same activation patterns selecting 35% threshold of most discriminative voxels with FDR corrected group maps representing 8 of 16 participants. The classification accuracy for each participant was always calculated with respect to the whole set of features that did not depend on the threshold chosen for the creation maps.

## Results

### Behavioral Study

**Figure 3:**
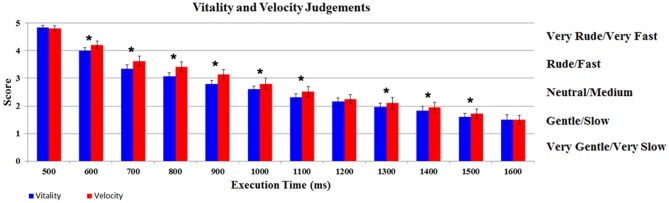
**Participants’ judgments relative to vitality and velocity tasks.** Graph shows for each level the score of the participants during vitality and velocity tasks. *Indicates the significant comparison between tasks relative to *post hoc* analysis for the interaction *Task × Execution Times* (*p* < 0.05 Newman Keuls corrected). The bars indicate the standard deviation (SD).

The participants’ judgments obtained during vitality and velocity tasks were automatically converted by E-Prime Software in numerical scores (very rude/very fast = 5; rude/fast = 4; neutral/medium = 3; gentle/slow = 2; very gentle/very slow = 1). The scores were then modeled using a GLM by a design matrix, comprising the participants’ score related to each task (vitality, velocity), for each execution time (12 levels). The results of the GLM analysis indicate a significant difference in judgments between the two *Tasks* (*F*_(1,17)_ = 10.07, *p* < 0.05, partial-*η*^2^ = 0.37, δ=0.85). More specifically, the mean score for velocity task (2.83; SD = 0.37) was shifted towards higher values relative to vitality task (2.66; SD = 0.31), in spite of the fact that the stimuli execution times were the same. In addition there was also a significant difference in the judgments of the *Execution Times* (*F*_(11,187)_ = 310.37, *p* < 0.05, partial-*η*^2^ = 1, δ=1). The interaction *Tasks* × *Execution Times* was also significant (*F*_(11,187)_ = 5.54, *p* < 0.05, partial-*η*^2^ = 0.90, δ = 0.89). *Post hoc* analysis revealed a significant difference between *Execution Times* comparisons [1–2 (500–600 ms), 2–3 (600–700 ms), etc., *p* < 0.05 Newman Keuls corrected]. As shown in Figure 3, for the interaction *Task × Execution Times*, *post hoc* analysis revealed a significant difference between vitality task and velocity task in nine different comparisons (600, 700, 800, 900, 1000, 1100, 1300, 1400, 1500 ms; *p* < 0.05 Newman Keuls corrected).

The analysis of the response times (RTs) revealed a difference between the two *Tasks* (*F*_(1, 17)_ = 13.8, *p* < 0.05, partial-*η*^2^ = 0.46, δ = 0.93) showing that participants were significantly faster in judging movement velocity (mean RT = 800 ms, SD = 220 ms) than vitality forms (mean RT = 980 ms, SD = 295 ms). In addition there was also a significant difference of RTs in the *Execution Times* (*F*_(11,187)_ = 4.3, *p* < 0.05, partial-*η*^2^ = 0.21, δ = 1).

A regression analysis was subsequently carried out to compare vitality and velocity judgment (dependent variable) as a function of the execution time (independent variable). As shown in Supplementary Figure 1, the best fit curve representing the relation between vitality perception and execution time follows a logarithmic trend (*R*^2^ = 0.94, *F* = 3060. *p* < 0.00). The same relationship was also observed for the velocity task (*R*^2^ = 0.87, *F* = 1513, *p* < 0.00). Taken together, these data indicate that the fitting of the vitality and velocity judgments as a function of the execution time, was very similar.

### fMRI Experiment

#### Response-Based Analysis Testing

This analysis was based on the participants’ responses (catch trials) that were indicated in the scanner using a response box during vitality and velocity tasks (see “Materials and Methods” Section). Within this analysis, we used the number of correct responses (hits, i.e., subjects correct responses to specific velocity or vitality, fast/rude—neutral/medium—gentle/slow) and RTs as dependent variables to assess possible effects of the two task difficulties. To this purpose, independent repeated measure GLM analyses, with two levels of task (vitality and velocity) and three levels of execution times (600, 1000, 1400 ms), were carried out. With respect to hits, the results revealed no difference between tasks (*p* > 0.05), showing that vitality and velocity were both judged correctly. On the contrary, the analysis of RTs revealed a difference between the two tasks (*F*_(1,15)_ = 7.7 *p* = 0.014, partial-*η*^2^ = 0.34, δ = 0.74) showing that participants were significantly faster in judging movement velocity (mean RT = 807 ms, SD = 116 ms) than vitality forms (mean RT = 907 ms, SD = 102 ms). The dissociation between accuracy and reaction time will be addressed in the discussion.

#### Univariate Analysis

##### Overall effect of “vitality” and “velocity” tasks

Observation of the video-clips for each task (*vitality* and *velocity*) vs. implicit baseline revealed a very similar activation pattern (Supplementary Figure 2). In particular, there was a signal increase in visual occipito-temporal areas, parietal lobe, SMA, premotor and prefrontal cortex (for statistical values and coordinates see Table 1). Additionally, insular activation was observed bilaterally. The direct contrast *vitality vs. velocity* tasks and *velocity vs. vitality* tasks, revealed no significant activations (*p* > 0.05). Also the GLM analysis performed on the insula did not reveal a significant effect of task (Left insula, *t*_(15)_ = 0.719, *p* = 0.48, Right insula, *t*_(15)_ = −0.618, *p* = 0.53).

**Table 1:** **Cerebral activity during (A) *vitality forms* vs. baseline; (B) *velocity* vs. baseline**.

	Left hemisphere				Right hemisphere			
Anatomical region	*x*	*y*	*z*	*t*	*x*	*y*	*z*	*t*
**(A) Vitality forms vs. Baseline**
Corpus callosum	−10	−26	24	14.7
Medial frontal gyrus	−7	16	42	10.5
Middle frontal gyrus	−37	46	15	7.9	38	46	15	4.9
Supramarginal gyrus	−52	−41	30	6.4
Superior frontal gyrus	−13	−5	63	5.4	29	43	9	4.9
Middle temporal gyrus	−49	−44	0	5.4
Precuneus					2	−50	51	5.6
Inferior frontal gyrus					50	37	3	5.3
Cerebellum					53	−56	−24	6.1
**(B) Velocity vs. Baseline**
Middle occipital gyrus	−22	−89	15	15.7
Cingulate gyrus	−10	13	42	9.8
Cerebellum	−10	−56	−33	6.3	53	−53	24	6.4
Middle frontal gyrus	−34	40	18	5.8	35	34	27	5.5
Middle temporal gyrus	−49	−44	3	5.8
Precentral gyrus	−25	−11	48	5.7
Inferior frontal gyrus	−49	7	30	5.6
Precuneus					5	−53	42	6.5
Fusiform gyrus					44	−32	−12	6.1
Post central gyrus					35	−20	30	6.0
Superior frontal gyrus					23	55	12	5.7
Thalamus					17	−11	15	5.6

*Local maxima, as shown in “Supplementary Figure 2”, are given in Talairach brain coordinates, significant threshold has been set at p_FDR_ < 0.05*.

##### Contrasts between vitality forms levels and velocity levels

All the direct contrasts within vitality task *(Rude vs. Gentle, Rude vs. Normal, Gentle vs. Normal, etc.,)* and velocity task* (Fast vs. Slow; Fast vs. Medium; Slow vs. Medium, etc.,)* did not reveal a significant activation pattern.

#### Multivariate Pattern Analysis

**Figure 4:**
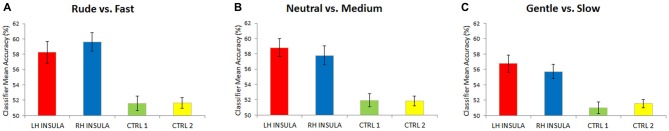
**Mean classification accuracy for 16 participants.** Accuracies obtained for the contrasts: *rude vs. fast* **(A)**, *neutral vs. medium* **(B)**, *gentle vs. slow* **(C)**. Accuracies were significantly different respect to the chance level (50%) only in the left and right insula. Differently, in each contrast level, control areas (CTRL 1, CTRL 2) not differ significantly from chance (50%).

The multivoxel pattern analysis revealed that the classifier mean accuracy for the levels across 16 participants was, for the left and right insula, respectively: left 58.2% (Wilcoxon, one sided; *p* < 0.01) and right 59.6% (*p* < 0.01) for the contrast *rude vs. fast*; left 58.8% (*p* < 0.01) and right 57.7% (*p* < 0.01) for the contrast *neutral vs. medium*; left 56.7% (*p* < 0.01) and right 55.7% (*p* < 0.01) for *gentle vs. slow* (Figure 4). For the two control areas (CTRL 1, CRTL 2), the classifier mean accuracy across the same 16 participants was respectively: 51.5% (*p* > 0.05) and 51.6% (*p* > 0.05) for the contrast *rude vs. fast*; 51.9% (*p* > 0.05) and 51.8% (*p* > 0.05) for the contrast *neutral vs. medium*; 50.9% (*p* > 0.05) and 51.5% (*p* > 0.05) for *gentle vs. slow*, that is chance level (Figure 4).

**Figure 5:**
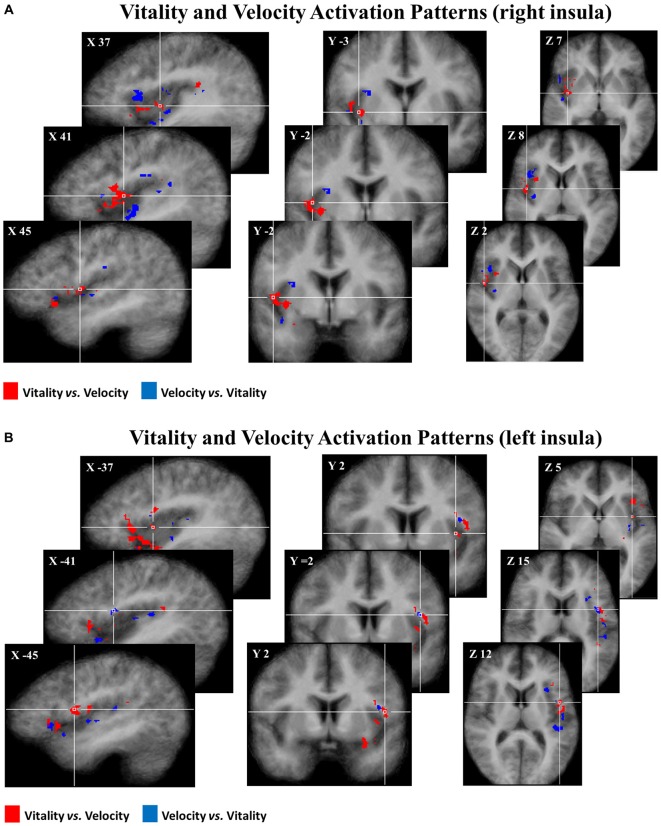
**Maps group of 50% of most discriminative voxels for the perceptual difference of *vitality* forms (red) and *velocity* (blue) collapsing three different contrasts (*rude vs. fast, neutral vs. medium, gentle vs. slow*) in the right (A) and in the left (B) insula.** Each voxel was reported if it was present in at least 10 of the 16 participants. These activation patterns (*p*_FDR_ < 0.05) are overlaid on the average anatomical template of 16 participants in Tailarach coordinates.

Subsequently, group discriminative maps were constructed and inspected for consistency of spatial activation patterns across participants. Figure 5 shows the pattern of discriminative voxels clustered in the insula. The red color indicates positive weights, corresponding to voxels that were more selective for vitality tasks with respect to velocity tasks, while the blue color indicates negative weights corresponding to voxels that were more selective for velocity tasks with respect to vitality tasks. In the discriminative maps, the three different comparisons (*rude vs. fast, neutral vs. medium, gentle vs. slow*) were collapsed together.

In addition, the multivoxel pattern analysis revealed that within each task, the classifier mean accuracy for the comparisons among vitality forms levels (i.e., *rude vs. gentle*, etc.) and velocity task (i.e., *fast vs. slow*, etc.) did not reach significance (*p* > 0.05) (right insula: *rude vs. gentle*, 52%, *fast vs. slow*, 51.9%; left insula: *rude vs. fast*, 51.8%, *fast vs. slow*, 50.7%).

## Discussion

In his seminal book on mother-infant relationship, Stern (1985) stressed that besides the goal and the intention of the performing agent, there is a third, fundamental aspect that an observer captures when viewing the actions of another individual: the action vitality forms. Vitality forms characterize how an action is performed and are detected on the basis of movement properties.

The aim of the present study was to assess whether action velocity, one of the crucial elements for understanding vitality forms, is encoded in the insula as such, or velocity triggers the insula neural populations encoding vitality forms. To this purpose we used multi-voxel pattern analysis (MVPA) with the aim to establish whether in the insula there are voxels discriminating vitality forms from velocity processing. Before performing the fMRI experiment, we carried out a behavioral study in which we presented arm actions performed at 12 different velocities. The task of the participants was to judge either the velocity or the vitality form of these actions. The results showed that, although the stimuli presented in the two tasks were identical, a significant difference was present in the subjects’ judgment according as to whether they were required to classify the observed actions for their vitality form or their velocity. This should indicate that the vitality form and velocity processing are two different perceptual constructs. In accord with this conclusion are also the reaction times results indicating that velocity processing was significantly faster than vitality processing (mean velocity RT: 800 ms; mean vitality form RT: 980 ms).

The neural bases of this finding are most likely due to the different circuits that mediate the two tasks. A previous study (Di Dio et al., 2013) investigated the neural correlates of velocity processing during the observation of actions performed by a biological effector (forelimb). The results showed that the circuit included, beside visual-occipito temporal areas and in particular MT/V5 and V6, a sector of the superior parietal lobule, extending towards the intraparietal sulcus, and the premotor cortex. As far as the insula is concerned there was an activation of the rostralmost part of it, known to be involved in cognitive tasks (Kurth et al., 2010), but not of the dorso-central part of the insula encoding vitality forms. It is likely therefore that this cortical circuit, which was found to be also activated in the present experiment, was responsible for the fast RTs during the velocity task. In contrast, the necessity to involve the dorso-central insula and to transform the velocity information into vitality forms, required an additional time and was therefore most likely responsible for longer RTs during vitality task.

On the basis of the behavioral study, we also selected three actions, corresponding to fast/rude (execution time: 600 ms; mean velocity: 1.06 m/s), medium/neutral (execution time: 1000 ms; mean velocity: 0.57 m/s) and slow/gentle (execution time: 1400 ms; mean velocity: 0.38 m/s) velocity/vitality judgments and used them for the fMRI study.

The multivoxel pattern analysis revealed the presence of discriminative voxels preferring vitality forms relative to velocity in the dorso-central sector of the insula especially in the right hemisphere. Our findings that the dorso-central part of the insula contains voxels discriminating vitality forms are in agreement with recent findings on the general functional organization of the insula in monkeys and humans. More specifically, monkey experiments in which the insula organization was studied by intracortical electrical stimulation showed that the insula consists of different sectors endowed with specific functional properties. The stimulation of the rostral sector of insula determines positive ingestive behavior dorsally, and negative ingestive behavior (e.g., disgust) ventrally (Jezzini et al., 2012). In contrast, the stimulation of the dorso-central sector of insula, which most likely corresponds to the part activated in the present experiment, elicits body parts movements with a rich representation of the movements of the upper limb.

A somehow similar organization pattern has been reported by Kurth et al. (2010) in humans in a meta-analysis based on a very large number of functional neuroimaging experiments. These authors described four main distinct functional fields in the human insula: the cognitive field, the sensorimotor, the olfactory-gustatory and the socio-emotional. Except for the cognitive field that is not clear in the monkey, there is a good correspondence in the two species between the other fields. The sensorimotor field appears to correspond to the sector involved in vitality form observation and production (Di Cesare et al., 2013, 2015). In contrast, the rostral part of the insula and its ventral part are related to classical Darwinian emotions (see on this point Dolan, 2002; Phillips et al., 2003; Wicker et al., 2003; Singer et al., 2004; Pichon et al., 2009). This functional characterization is in accord with the view of Stern mentioned above that there is a fundamental difference between vitality forms and the classical Darwinian emotions.

Some very recent findings showed that the dorso-central insula is involved in both vitality form execution and recognition suggesting that neurons of this sector of the insula could be endowed with the mirror mechanism (Di Cesare et al., 2015). An interesting question concerns the output of the dorsal-central insula and how this output may modulate the cortical circuits underlying voluntary movements. A possible answer to this question may come from some anatomical data obtained in the monkey. It has been recently shown that the dorso-central sector of the insula has rich connections with the parietal and frontal areas that form the circuit involved in the organization of arm movements in the monkey (Jeannerod et al., 1995; Nelissen and Vanduffel, 2011) and namely with areas AIP (Borra et al., 2008), F5 (Gerbella et al., 2011), and 12r (Borra et al., 2011). It is important to stress that a homologous parieto-frontal circuit underlies arm movement organization also in humans (Rizzolatti et al., 2014).

In agreement with these findings, showing a connection between insula and parieto-frontal circuit, are also the results of Almashaikhi et al. (2014a,b) who stimulated electrically the middle and posterior short gyri of the insula in patients with drug-resistant epilepsy. The data showed that the stimulation of these insular sectors determines evoked potential in the precentral gyrus and the superior and inferior parietal lobules. These findings confirm the connectivity of these sectors of the insula with the cortical areas involved in the control of the voluntary movements as anatomically demonstrated in the monkey.

In conclusion, the main finding of our study is the demonstration that the insula is a key area for vitality forms processing. During social interactions, this area is triggered by the physical aspects of an observed action determining in the observer a communicative/affective construct (vitality form). In virtue of this mechanism, the observer is able to understand the others’ internal state. As shown recently by Di Cesare et al. (2015), this mechanism is also involved in vitality form production (i.e., action execution) allowing an individual to communicate his/her affective internal state to others.

## Author Contributions

GDiC performed research; GDiC, ER and GV analyzed data; GDiC, GV and GR wrote the article.

All authors listed, have made substantial, direct and intellectual contribution to the work, and approved it for publication.

## Funding

This study was supported by European Research Council (ERC) “COGSYSTEM” project no. 250013 (to GR) and Fondazione Cassa di Risparmio di Parma (CARIPARMA).

## Conflict of Interest Statement

The authors declare that the research was conducted in the absence of any commercial or financial relationships that could be construed as a potential conflict of interest.

## Supplementary Material

Supplementary Figure 1 **Regression graphs.** Graphs depict the logarithmic relation between participants’ judgments during the tasks [vitality task **(A)**, velocity task **(B)**] and action execution time (ms). For each execution time, points indicates participants mean score (very rude/very fast = 5; rude/fast = 4; neutral/medium = 3; gentle/slow = 2; very gentle/very slow = 1). The velocity peak corresponding to each judgment is reported on the right side.

Supplementary Figure 2 **Signal change during (A) *vitality* task vs. *implicit baseline* and (B) *velocity* task vs. *implicit baseline* (fixation cross).** These activations (P_FDR_ < 0.05) are rendered into a Talairach brain template.
