# Supplementary figures and images for: Vitality Forms Processing in the Insula during Action Observation: A Multivoxel Pattern Analysis
Source: Front Hum Neurosci. 2016 Jun 9;10:267. doi: 10.3389/fnhum.2016.00267 (PMC4899476; doi:10.3389/fnhum.2016.00267)

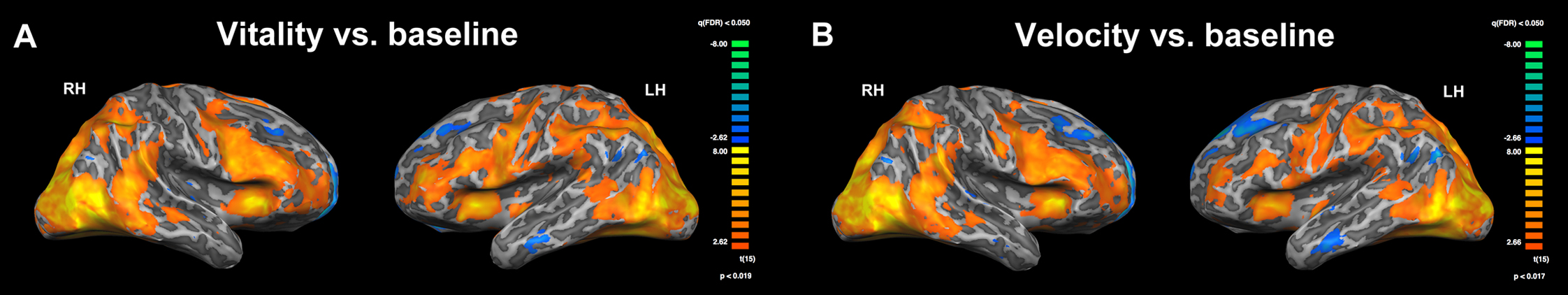

Supplement: Supplementary Figure 1 — Regression graphs. Graphs depict the logarithmic relation between participants’ judgments during the tasks [vitality task (A), velocity task (B)] and action execution time (ms). For each execution time, points indicates participants mean score (very rude/very fast = 5; rude/fast = 4; neutral/medium = 3; gentle/slow = 2; very gentle/very slow = 1). The velocity peak corresponding to each judgment is reported on the right side. [file Image_1.jpeg]

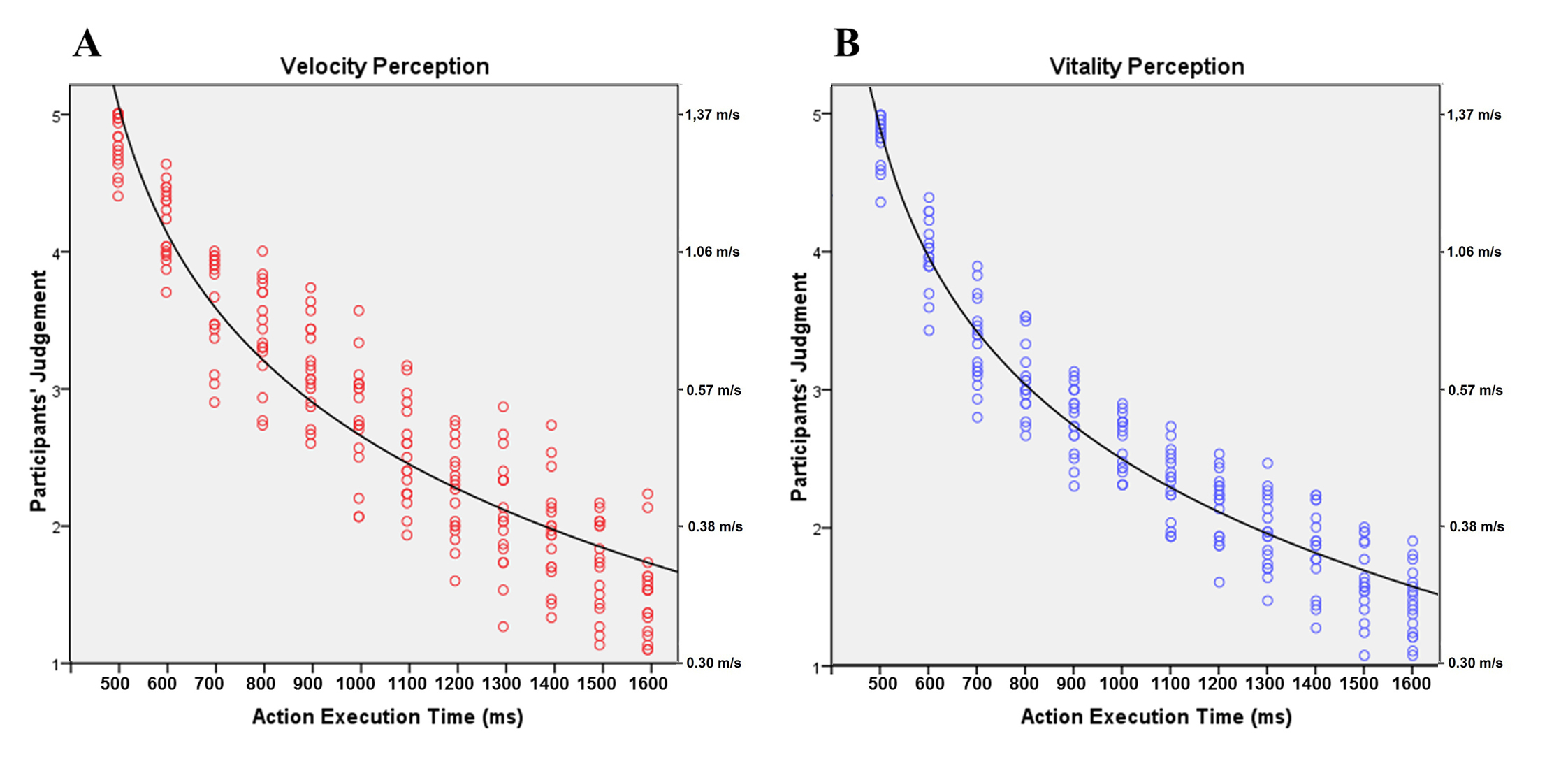

Supplement: Supplementary Figure 2 — Signal change during (A) vitality task vs. implicit baseline and (B) velocity task vs. implicit baseline (fixation cross). These activations (PFDR < 0.05) are rendered into a Talairach brain template. [file Image_2.jpeg]
